# Subfecundity and associated factors among pregnant mothers receiving antenatal care at public health facilities in Ambo town Oromia region, Ethiopia: a cross-sectional study

**DOI:** 10.3389/fgwh.2025.1506481

**Published:** 2025-07-10

**Authors:** Elias Andesha, Gizachew Abdissa, Gemechu Ganfure, Melese Adugna, Merga Sheleme, Jemal Bedane

**Affiliations:** ^1^ Department of Midwifery, School of Health Science, Ambo University Waliso Campus, Waliso, Ethiopia; ^2^ Department of Midwifery, College of Medicine and Health Science, Ambo University, Ambo, Ethiopia; ^3^ Department of Paediatric Nursing, College of Medicine and Health Science, Ambo University, Ambo, Ethiopia; ^4^ School of Medicine, College of Health and Medical Science, Haromaya University, Harar, Ethiopia; ^5^ Department of Midwifery, College of Medicine and Health Science, MadaWalabu University, Shashamane Campus, Shashamane, Ethiopia

**Keywords:** sub-fecundity, waiting time to pregnancy, public health facilities, Ambo, Ethiopia

## Abstract

**Background:**

Subfecundity is defined by a time to pregnancy of more than 12 months with unprotected sexual intercourse. Despite many couples experiencing psychological, social, and economic effects as a consequence of subfecundity, it has been inadequately explored in Ethiopia.

**Objective:**

Since there is limited information available in Ethiopia on subfecundity and no further studies have been conducted in the study area, this study will serve as input. Therefore, this study aimed to assess the magnitude of subfecundity and associated factors in Ambo town.

**Methods:**

A cross-sectional study was employed using systematic sampling to select 368 pregnant mothers. Data were collected through face-to-face interviews using a pre-tested structured questionnaire supplemented with a review of medical records. Bivariate and multivariable logistic regression were performed to identify factors associated with subfecundity. The statistical significance was declared using 95% CI, with a *p*-value <0.05.

**Result:**

A total of 361 mothers (21.3%, 95% CI: 17.20–25.50) were interviewed, resulting in a response rate of 98%. Subfecundity was more likely among mothers aged >35 years (AOR = 3.74, 95% CI: 1.38–10.18), menstrual cycle irregularities (AOR = 3.15, 95% CI: 1.66–5.98), those whose coital frequency was 1 day per week (AOR = 4.77 95% CI: 2.22–10.23), mothers with primigravida (AOR = 2.29, 95% CI: 1.18–4.41), those who used contraceptives (AOR = 1.87, 95% CI: 1.02–3.50), and those who were stressed before conceiving (AOR = 1.95, 95% CI: 1.03–3.70).

**Conclusion:**

This study found that the prevalence of subfecundity was 77% (21.3%, 95% CI: 17.2–25.5), which is slightly higher than previous findings in Ethiopia. Subfecundity was more likely among mothers age >35, those with primigravida, mothers who experienced menstruation irregularities, those whose coital frequency was less than twice per week, mothers using an injectable contraceptive method, and those who were stressed before the current pregnancy. Thus, health professionals should provide information for women at preconception care clinics, sexual and reproductive health clinics, and family planning clinics to those who wish to become pregnant before the age of 35 years to increase the frequency of coital practice, decrease stress, and encourage treatment for menstruation irregularities.

## Background

Fecundity is the physiological capacity to have children, which occurs in the span between menarche and menopause in women refers to the physical capability to have a child (1). Fecundability, which is the monthly likelihood of pregnancy, is about 20% among fertile couples. Subfecundity is defined by a time to pregnancy of more than 12 months with unprotected sexual intercourse, i.e., when women have certain physical conditions making it possible but unlikely to conceive (2).

In epidemiological studies, waiting time to pregnancy (WTTP) measures how long a couple takes to conceive and provides an estimation of the likelihood of conceiving a clinically observable pregnancy per cycle, i.e., it is used as a measure of a couple’s fecundity (3–5). Epidemiological studies categorize fecundity status using WTTP (with fecundity within 12 cycles/months and sub-fecundity after 12 months of waiting time to conceive) (6–8). As WTTP increases, the probability of conceiving within one year decreases (9). A prolonged WTTP represents a daily problem for couples (10). Being unable to conceive for a prolonged time can cause friction between couples (11, 12). Waiting for months/years is a risk factor for a breakdown in the relationship, finding another individual to cohabit with, sexually transmitted infections (STIs), separation, dependence, psychological suffering, social stigmatization, hopelessness, worry, negative economic consequence, disrespect from husband and relatives, and neglect by in-laws, t (11).

Globally, from 2009 to 2015, around 60 to 80 million people a year experienced an inability to get a wanted pregnancy (8, 13). As a result, couples are forced to keep up the number of months/years without pregnancy to conceive a baby (13, 14). The average possibility of pregnancy after one year (subfecundity) is around 85%. In Europe, a waiting time for pregnancy greater than 12 months ranges from 10.4% to 17.58% (15, 16). The 2016 South African report of subfecundity found it to be 22% (17). The 2017 demographic and health review report of low- to middle-income countries indicated that subfecundity was 31.1% (13). In 2018 and 2020, studies conducted in Addis Ababa and Arba Minch indicated that a waiting time for pregnancy of about 12 months was 18.3% and 17.8%, respectively (6, 18).

Identifying subfecundity its and associated factors is important for individuals and health care providers to provide information and enhance awareness on the prevention and treatment of subfecundity, as well as coping strategies (16). According to some evidence, there are many different factors that may be associated with subfecundity. Socio-demographic factors such as the age of the couple are related to WTTP (19, 20). Reproductive health-related factors are also linked, for example, abortion is associated with the subjective time to conceive (21). The irregularity of the menstrual cycle and the use of injectable contraceptive methods before pregnancy are also factors associated with subfecundity (22). Substance use, such as smoking cigarettes, is related to increased waiting time to conceive (23, 24). Alcohol, chewing, and coffee drinking are also identified as factors related to subfecundity (6, 25). Lifestyle, exercise, psychological stress, and environmental pollutants are factors related to subfecundity (26).

Despite allocating resources for women, especially during pregnancy and lactation, such as advising on prevention and treatment in line with reproductive health and childbearing and counselling reproductive-age women to prevent factors causing delayed time to conceive before starting assisted reproductive technology (ART), subfecundity is still a major health problem for couples (27). Thus, in this study, we aimed to assess the magnitude of subfecundity by using WTTP and intended to address variables that are associated with subfecundity like stress, physical activity, and exposure to environmental and occupational chemicals and pollutants, and provide more information and awareness about subfecundity to overcome its prevalence among mothers receiving antenatal care in Ambo town public health facilities.

## Methods

### Study settings

Ambo, which was established in 1889 and is located 114 km to the west of the capital Addis Ababa, is one of the towns in central Ethiopia. According to the Ambo Town Health Administration office in 2014, the total population of the town is 97,317, of which 49,602 are males and 47,715 are females. Among those females, 10,974 are in the reproductive age group and 395 of them are pregnant. In the town, there is one referral hospital, one general hospital, two health centers, and 21 medium private clinics. The study was conducted in Ambo town, from 1st August to September 30 of 2022.

### Study design and population

A facility-based cross-sectional study design was conducted. All pregnant mothers who had attended an antenatal care (ANC) service at Ambo town public health facilities were the source population, while pregnant mothers who had attended an ANC service at Ambo town public health facilities during the study period were the study population. All randomly selected pregnant mothers during the study period were the study units.

### Inclusion criteria

All pregnant mothers who had attended ANC services were included in the study.

### Exclusion criteria

Pregnant mothers with any medically assisted reproduction technology including Assisted Reproductive Therapy, those who were seriously ill, and those who had mental problems were excluded from the study.

### Sample size determination and sampling techniques

The sample size for the first objective was determined by using a single population proportion formula using the proportion of subfecundity from the study conducted in Arba Minch, which was 17.8% (*P* = proportion of subfecundity = 0.178, *Z*(1 − *α*/2) = critical value for 95% confidence level, which equals to 1.96, with a 10% non-response rate, meaning the final sample size was 248) (18). Also, the sample size was calculated for factors associated with subfecundity using Epi-Info version 7.2.4.0 Stat Calc. computer software using assumptions like power 80%, confidence interval 95%, and percent of cases among exposed and unexposed individuals. Ultimately, the sample size calculated from the partner's age before the pregnancy was larger than the sample size for the first objective and taken as the final sample size of the study, which is 368 pregnant women with a 10% nonresponse rate. Last year, booked appointments in August and September for ANC in Ambo University Referral Hospital, Ambo General Hospital, Ambo Health Centre, and Awaro Health Centre were 351, 242, 235, and 204, respectively, and the total number of booked ANC appointments of all public health facilities was 1,032. So, the required sample size for each facility was allocated proportionally to all facilities based on their monthly booked ANC report figures by using the following formulas

$$\mathrm{The}\;\mathrm{SS}\;\mathrm{for}\;\mathrm{each}\;\mathrm{Facility}=\frac{\mathrm{Monthly}\;\mathrm{booked}\;\mathrm{ANC}\;\mathrm{reports}\;\mathrm{from}\;\mathrm{each}\;\mathrm{facility}*\mathrm{final}\;\mathrm{SS}}{\mathrm{Total}\;\mathrm{numbers}\;\mathrm{of}\;\mathrm{monthly}\;\mathrm{booked}\;\mathrm{ANC}\;\mathrm{reports}\;\mathrm{all}\;\mathrm{facilities}}$$

Where SS is sample size.

Therefore, the sample size for Ambo University Referral Hospital = 351 × 368/1,032 per month = 125, for Ambo General Hospital = 242 × 368/1,032 per month = 86, for Ambo Health Centre was 235 × 368/1,032 = 84 and for Awaro Health Centre was 204 × 368/1,032 = 73. Participants were chosen from each facility according to the above sample size.

Finally, the required number (*n* = 368) of participants were selected using a systematic random sampling technique by using an interval (K^th^) calculated from the monthly follow-up of pregnant mothers divided by the number of allocated samples to the health facilities. The first study unit was selected randomly, and then every third interval was selected for face-to-face interviews and assisted questionnaires when leaving the ANC.

### Data collection

Data were collected through interviews of eligible participants using a structured questionnaire and supplemented with a review of their medical records, which was adapted by reviewing different literature of the same purpose (18, 26, 28). The tool consisted of five parts: sociodemographic characteristics of the mother, sexual and reproductive health, substance use, medical problems, and lifestyle characteristics. Data were collected by four BSc midwives and supervised by two MSc midwives in maternity nursing and reproductive health who received training on data collection and were fluent in the local language.

To ensure the quality of data, properly designed data collection instruments were prepared. Firstly, the tool was prepared in English and translated into the Afan Oromo language by a language expert and translated back to English to certify the consistency of meaning. Before data collection, pre-testing was conducted in a 5% population among pregnant mothers attending ANC services at Guder Health Center. On-site daily supervision was carried out throughout the data collection period.

### Variables of the study and measurement

The dependent variable of the study, subfecundity, was measured as fecundity status; it was categorized “Yes” if fecund, “No” if not, and coded “1” and “0” respectively. Those categorized as “No” were considered as subfecund and re-coded “1”; while those categorized as “Yes” were considered as non-subfecund and re-coded “0”. The independent variables included socio-demographic conditions (age, marital status, education, mother's working hours, occupation, family income, and duration of cohabiting), reproductive health-related conditions (parity, abortion before the current pregnancy, frequency of sexual intercourse before the current pregnancy, menstrual pattern before the current pregnancy, and type of contraceptive used before the current pregnancy), substance-use-related factors before the current pregnancy (coffee intake, smoking cigarettes, alcohol drinking, and khat chewing), lifestyle factors before the current pregnancy (exercise, psychological stress, and environmental pollutants), and medical-related conditions before the current pregnancy (HIV and Diabetes mellitus).

### Operational definitions

***Waiting time to pregnancy (WTTP*):** the number of months or years that a couple waits to conceive a pregnancy by performing unprotected sexual intercourse and excluding the months of sexual abstinence due to illness, distance due to work, or other causes. In this study, WTTP was used to identify the fecundity status of a mother with naturally planned conception and was dichotomized as “Subfecundity” (WTTP greater than 12 months) and “Fecundity” (WTTP for 12 and below months) (29, 30).

***Fecundity status***: the physiological capacity to have children, which occurs in the period between menarche and menopause in women (1, 31).

***Natural Conception*:** Pregnancy without Assisted Reproductive Technology (ART).

### Statistical analysis

All collected data were cross-checked for completeness and consistency, coded, double-entered to Epi-Data 3.1, and exported to SPSS 26 for analysis. Descriptive statistics, binary, and multivariable logistic regression analysis were used to identify associated factors. Variables having a *P*-value <0.25 in the bivariable logistic regression analysis were selected for the multivariable logistic regression model for adjustment of confounding effect between independent variables and in order not to miss important variables. Multicollinearity was checked using variance inflation factor (VIF) or the tolerance test, and no multicollinearity was detected. Model fitness was also checked using Hosmer-Lemeshow tests, and the model was well-fitted. Association was described using an adjusted Odds Ratio (AOR) along with a 95% confidence interval (CI). Finally, *p*−<0.05 in the multivariable analysis was considered as a cut-off point for a statistically significant association.

## Results

### Characteristics of study participants

In this study, of a total of 368 approached mothers, 361 (98%) participated. The mean age of respondents was 26 ± 5.486 years. The majority of the respondent mothers were married 348 (96.4%). Of the mothers, 165 (45.7%) attended college or above. Eighty-eight (24.4%) of respondents were government employees (Table 1).

**Table 1:** Sociodemographic characteristics of pregnant mothers attending antenatal care services in public health facilities of Ambo town, Oromia region, Ethiopia (*n* = 361).

Variables	Categories	Frequency (n)	Percent (%)
Mothers age	<25	173	47.9
	25–35	157	43.5
	>35	31	8.6
Partners age (*n* = 350)	<25	62	17.7
	25–35	205	58.6
	>35	83	23.7
Educational status of the mother	No formal education	29	8.0
	Primary education	86	23.8
	Secondary education	81	22.4
	Colleges and above	165	45.7
Educational status of partner (*n* = 350)	No formal education	5	1.4
	Primary education	55	15.7
	Secondary education	95	27.2
	Colleges and above	195	55.7
Occupational status of the mother	Housewife	161	44.6
	Merchant	65	18.0
	Student	30	8.3
	Government Employee	88	24.4
	Daily laborer	11	3.0
	Other*	6	1.7
Average working hours per week of mother	20–40	51	14.1
	41–60	194	53.7
	61–80	48	13.3
	≥81	68	18.8
Occupational status of partner (*n* = 350)	Farmer	32	8.8
	Merchant	67	18.6
	Student	13	3.6
	Government Employee	165	45.7
	Daily laborer	67	18.6
	Other**	17	4.7
Average working hours per week of partner (*n* = 350)	20–40	48	13.73
	41–60	166	47.43
	61–80	53	15.1
	≥81	83	23.7
Marital status	Unmarried (cohabiting)	11	3.0
	Married	350	97.0
Duration of months they live together (*n* = 350)	1–60	207	59.1
	61–120	84	24.0
	≥121	59	16.9
Religion of respondent	Orthodox	138	38.2
	Protestant	176	48.8
	Muslim	9	2.5
	Catholic	12	3.3
	Wakefata	15	4.2
	Other***	11	3.0
Average family income	≤2,000	29	8.0
	2,001–4,000	63	17.5
	4,001–6,000	102	28.3
	>6,001	167	46.3
Residential area	Rural	57	15.8
	Urban	304	84.2

Other* Waiter, Other** Driver, Other*** Adventist, Hawariyat.

Among the respondents, almost half, 165 (45.7%), were in the second trimester, and 186 (51.5%) were primigravida. About 86 (23.8%) of mothers had a coital frequency of one day per week (Table 2).

**Table 2:** Reproductive health-related characteristics of pregnant mothers attending antenatal care services in public health facilities of Ambo town, Oromia region, Ethiopia (*n* = 361).

Variables	Categories	Frequency (*n*)	Percent (%)
Current gestational age	<13	35	9.7
	13–27	165	45.7
	28–40	160	44.3
Gravidity	Primigravida	186	51.5
	Multigravida	175	48.5
Parity	Primipara	88	24.4
	Multipara	87	24.1
	Nulipara	186	51.5
History of Abortion	Yes	38	10.5
	No	323	89.5
Menstruation status	Regular	295	81.7
	Irregular	66	18.3
Coital frequency per week	1	86	23.8
	2–3	160	44.3
	>3	115	31.9
Type of contraceptive used	None	137	38.0
	Condom	14	3.9
	Oral contraceptive	40	11.1
	Injection	97	26.9
	Implant	63	17.5
	IUCD	10	2.8

Among the respondents, 192 (53.2%) drank four cups of coffee or more per day. 122 (33.8%) mothers had a history of drinking alcohol before the current pregnancy. 16 (4.4%) of the mothers’ partners had a history of smoking cigarettes every day before the current pregnancy (Table 3).

**Table 3:** Substance-use-related characteristics of pregnant mothers attending antenatal care services in public health facilities of Ambo town, Oromia region, Ethiopia (*n* = 361).

Variables	Categories	Frequency (*n*)	Percent (%)
Mothers drinking coffee per day	None	8	2.2
	1 cup	31	8.6
	2 cups	72	19.9
	3 cups	58	16.1
	≥4 cups	192	53.2
Partners drinking coffee (*n* = 350)	None	21	6.0
	1 cup	68	19.4
	2 cups	127	36.3
	3 cups	59	16.6
	≥4 cups	76	21.7
Mothers chewing khat	Yes	5	1.4
	No	356	98.6
Partners chewing khat (*n* = 350)	Yes	34	9.7
	No	316	90.3
Mothers drinking alcohol	Yes	122	33.8
	No	239	66.2
Partners drinking alcohol (*n* = 350)	Yes	159	45.4
	No	191	54.6
Mothers smoking cigarettes	Yes	1	0.3
	No	360	97.7
Partners smoking cigarettes (*n* = 350)	Yes	16	4.6
	No	334	95.4

The majority of respondents 340 (94.2%) knew their HIV status before the current pregnancy. Of the total respondents, 242 (67%) performed exercise during their daily activities. About 20.3% of respondent mothers were exposed to some environmental and occupational chemicals and pollutants (Table 4).

**Table 4:** Medical and lifestyle characteristics of pregnant mothers attending antenatal care services in public health facilities of Ambo town, Oromia region, Ethiopia (*n* = 361).

Variables	Categories	Frequency (*n*)	Percent (%)
Mother HIV status	Positive	3	0.8
	Negative	337	93.4
	Unknown	21	5.8
Partner HIV status	Positive	3	0.8
	Negative	292	80.9
	Unknown	66	18.3
Mother medical problem	None	344	95.3
	DM	2	0.6
	Other*	15	4.2
Partner medical problem (*n* = 350)	None	336	96.0
	DM	1	0.3
	Other**	13	3.7
Type of sport/physical activity	None	58	16.1
	Running	47	13.0
	Riding bicycle	13	3.6
	Weight lifting	5	1.4
	Other***	242	67.0
Stress or anxiety	None	180	49.9
	In work	48	13.3
	In private	43	11.9
	In love	29	8.0
	Other****	61	16.9
Occupational chemicals or pollutants	None	289	80.1
	Radiation	31	8.6
	Pesticide	19	5.3
	Welding	19	5.3
	Other*****	4	1.1

*,** Indicates Asthma, Gastritis, Kidney disease, Hypertension, *** Daily activities, ****Income, helplessness, *****other factors.

### Magnitude of subfecundity

**Figure 1:**
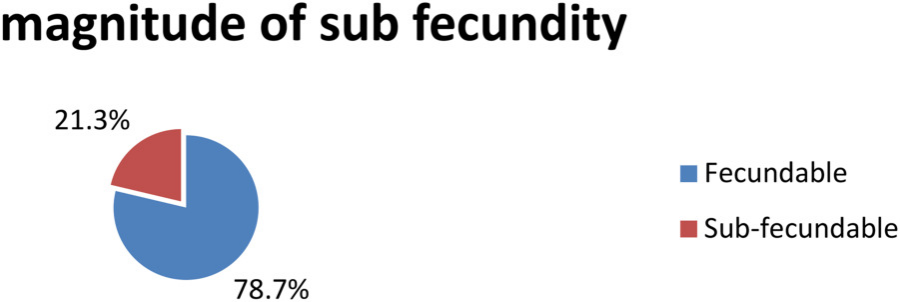
Magnitude of subfecundity among pregnant mothers attending antenatal care services in public health facilities of Ambo town, Oromia region, Ethiopia, 2021.

In this study, the magnitude of subfecundity among pregnant mothers attending antenatal care services at Ambo town public health facilities was 77% (21.3%, 95% CI: 17.2–25.5) (Figure 1).

### Factors associated with subfecundity

Before and after an adjustment was made, mother's age >35, menstruation irregularities, coital frequency per week, using an injectable contraceptive method, the first pregnancy, and mothers’ stress before the current pregnancy were significantly associated factors with subfecundity. The odds of subfecundity among mothers found in the age category of >35 years before the current pregnancy were 3.7 times (AOR = 3.74, 95% CI: 1.38–10.18) compared to mothers in the age category of <25 years. Mothers who had irregular menses before the current pregnancy were three times (AOR = 3.15, 95% CI: 1.66–5.98) more likely to be subfecund than mothers who had regular menses. The likelihood of subfecundity was increased by two times (AOR = 2.28, 95% CI: 1.18–4.41) among mothers becoming pregnant for the first time than mothers who had other children. Mothers with a coital frequency of once per week before the current pregnancy were 4.8 times (AOR = 4.77, 95% CI: 2.22–10.23) more likely to be subfecund than mothers who practiced greater than three times per week. Mothers who used an injectable contraceptive were nearly two times (AOR = 1.87, 95% CI: 1.02–3.50) more likely to be subfecund than those who did not use an injectable contraceptive. Mothers who were stressed were almost two times (AOR = 1.95, 95% CI: 1.03–3.70) more likely to be subfecund than those who had no stress (Table 5).

**Table 5:** Factors associated with Subfecundity among pregnant mothers attending antenatal care services in public health facilities of Ambo town, Oromia region, Ethiopia (*n* = 361).

Variables	Categories	Fecundity status		cOR (95% CI)	aOR (95% CI)	*P*-value
		Yes (%)	No (%)			
Age of mother	>35	12 (38.7)	19 (61.3)	3.01 (1.32–6.85)	3.74 (1.37–10.18)	.010*
	25–35	35 (22.3)	122 (77.7)	1.37 (0.79–2.36)	1.56 (0.81–3.01)	.186
	<25	30 (17.3)	143 (82.7)	1	1	
Pregnancy for the first time	Yes	48 (26.0)	137 (74.0)	1.78 (1.06–2.97)	2.29 (1.183–4.41)	.014*
	No	29 (16.5)	147 (83.5)	1	1	
Menses’ regularity	Irregular	29 (44.0)	37 (56)	4.03 (2.27–7.18)	3.20 (1.6–5.98)	.001*
	Regular	48 (16.3)	247 (83.7)	1	1	
Coital frequency (days per week)	1	36 (41.9)	50 (58.1)	4.8 (2.40–9.60)	4.76 (2.2–10.23)	.001*
	2–3	26 (16.3)	134 (83.7)	1.29 (0.65–2.57)	1.52 (0.73–3.19)	.265
	>3	15 (13.0)	100 (87)	1	1	
Use of Injectable Contraceptive Methods	Yes	55 (24.5)	169 (75.5)	1.70 (0.98–2.90)	1.87 (1.01–3.50)	.049*
	No	22 (16.1)	115 (83.9)	1	1	
Mother drinking alcohol	Yes	34 (27.9)	88 (72.1)	1.76 (1.05–2.95)	1.20 (0.65–2.32)	.523
	No	43 (18.0)	196 (82.0)	1	1	
Partner medical problem	Yes	7 (50.0)	7 (50.0)	3.96 (1.34–11.65)	3.40 (0.89–12.80)	.071
	No	70 (20.2)	277 (79.8)	1	1	
Stress	Yes	52 (28.7)	129 (71.3)	2.50 (1.47–4.3)	1.95 (1.03_3.70)	.042*
	No	25 (13.9)	155 (86.1)	1	1	
Exposure to occupational chemicals or pollutants	Yes	25 (34.7)	47 (65.3)	2.42 (1.37–4.29)	0.90 (0.40–2.02)	.803
	No	52 (18.0)	237 (82.0)	1	1	

1, reference; COR, crude odds ratio; AOR, adjusted odds ratio.

* Indicates that significant at a *p*-value <0.05.

## Discussion

In this study, we assessed the prevalence of subfecundity and associated factors among pregnant mothers attending antenatal care services at Ambo town public health facilities. We found that the overall magnitude of sub-fecundity was 21.3% 95 CI: (17.20–25.50%). Our finding is a little higher than studies conducted in Addis Ababa (18.3%) and Arba Minch (17.8%) and a little lower than studies conducted in South Africa (22%) (7, 17, 18). A possible reason might be variations in the study population and the socioeconomic status of the population. The finding of subfecundity reported in this study is higher than the studies conducted in European regions (16.1%), Palestine (13.4%), and Germany (10.4%) (8, 32, 33). A possible reason might be variations in the study population, socioeconomic status, and health policy of the country. Also, the finding of subfecundity reported in this study is lower than a multicenter study of Asian countries (45%) (34). A possible reason for this might be a variation in the socioeconomic status of the study population.

In this study, subfecundity was found to be more likely among women who were aged >35, had menstruation irregularities, had low coital frequency per week, used an injectable contraceptive method, had their first pregnancy, and experienced stress before the current pregnancy. In line with several other studies, subfecundity was more likely among mothers aged >35 years before the pregnancy (7, 14, 22, 35, 36). A possible reason might be that, as age advances, the ovarian reserve and oocyte quality and quantity decline (37). The occurrences of subfecundity were more likely to increase among mothers becoming pregnant for the first time than among mothers who had experienced pregnancy. A possible reason may be a psychosocial adaptation to pregnancy and motherhood experiences for multigravida women. Consistent with other studies, women who had previously used injectable contraceptive methods had a significantly lower probability of conceiving early compared with women who did not use them (38). This might be due to the use of injectable contraceptive methods that may delay or prolong the return of the menstruation cycle and the effect of contraceptive use potentially compromising ovarian function.

In line with other studies, mothers who had sexual intercourse once per week were more likely to be subfecund than mothers who practiced sexual intercourse ≥3 days per week (18, 38). A possible explanation for this might be that when the timeof ovulation is mismatched with the time of sexual intercourse per week, couples might be unsure over the appropriate time to conceive. Consistent with prior studies, mothers who had irregular menstruation were also more likely to be subfecund (8, 10, 36, 38, 39). A possible reason may be due to ovulation day. When menstruation day is irregular, it is difficult to determine the day on which ovulation will occur, and the ability to get pregnant decreases.

In addition, mothers feeling stressed were more likely to be subfecund. This finding is in line with a prior study conducted in Cleveland, USA (40). It may be because stress can decrease libido, resulting in the couples having less frequent intercourse.

### Strengths and limitations of the study

A strength of this study is that social desirability bias might be reduced since data were collected by health professionals and the client and healthcare providers have trust in each other due to confidentiality. In addition, the use of medical record reviews might reduce response bias that might have otherwise occurred when the women were asked to respond to their HIV status. However, our study also has some limitations. First, since we limited our study to only public health facilities, private hospitals and clinics that gave delivery services were not included. Second, recall bias might have occurred when mothers were asked to recall their time to pregnancy, and this study was limited to subfecundity/subfertility, excluding the infertile women population.

## Conclusion

In this study, we found that the prevalence of subfecundity in the study area is high compared to the previous study. Consistent with the previous studies, subfecundity is more likely among mothers >35 years, mothers with menstruation irregularities, mothers engaging in sexual activity once per week, mothers experiencing stress, and those using an injectable contraceptive method. In addition, mothers who became pregnant for the first time were more likely to develop subfecundity. There is a need to improve women's awareness to plan pregnancy before the age of 35 years, increase the frequency of coital practice, decrease stress, and get treatment for menstruation irregularities to reduce sub-fecundity.

## Data Availability

The original contributions presented in the study are included in the article/Supplementary Material, further inquiries can be directed to the corresponding author.
