# Supplementary material for: Subfecundity and associated factors among pregnant mothers receiving antenatal care at public health facilities in Ambo town Oromia region, Ethiopia: a cross-sectional study
Source: Front Glob Womens Health. 2025 Jul 10;6:1506481. doi: 10.3389/fgwh.2025.1506481 (PMC12287065; doi:10.3389/fgwh.2025.1506481)
Supplement: Supplementary file 1 [file Table1.docx]

## English Version of Questionnaire

This questionnaire is prepared to assess the magnitude of sub-fecundity and associated factors among pregnant mothers attending ANC service in Ambo Town public health facilities.

**Interview and medical record review**

|  | | | | | | |
| --- | --- | --- | --- | --- | --- | --- |
|  | Questionnaire Code______ Date of Interview____________  Name and signature of interviewer**_______________________________** ________  Name and signature of Supervisor_______________________________ ________ | | | | | |
|  | **Instruction**: Circle the appropriate answer provided and where applicable write the required responses in the spaces provided. | | | | | |
| **S/No** | **Part I: Socio-demographic questionnaire** | | | | | |
| 101 | How old are you? | | -----years? | |  | |
| 102 | How old was your partner? | | 1. -----years? | |  | |
| 103 | What was your educational status? | | 1. 1. no formal education 2. 2. Primary education(1-8) 3. 3. Secondary 4. 4. colleges + | |  | |
| 104 | What was your partner’s educational status? | | 1. 1. no formal education 2. 2. Primary education(1-8) 3. 3. Secondary 4. 4. colleges + | |  | |
| 105 | What was your occupation? | | 1. 1. Housewife 2. 2. Merchant.3. Student 3. 4. Government Employee 4. 5. Daily laborer 5. 6. Other(specify)_________ | |  | |
| 106 | How many average working hours per week were you working? | | ________hrs/week | |  | |
| 107 | What was your partner’s occupation? | | 1. 1. Farmer 2. 2. Merchant. 3. 3. Student 4. 4. Government Employee 5. 5. Daily laborer 6. 6. Other(specify)_________ | |  | |
| 108 | How many average working hours per week was your partner working? | | ________hrs/week | |  | |
| 109 | What was your marital status? | | 1. 1. unmarried cohabiting partner, 2. 2. Married | |  | |
| 110 | For how long and for how many months did you live together? | | -------months? | |  | |
| 111 | What is your religion? | | 1. 1. Orthodox 2. 2. Protestant 3. 3. Catholic 4. 4. Muslim 5. 5. Wakefata/tu 6. 6. Others | |  | |
| 112 | How much is your family’s monthly income in ETB? | | --------ETB/Months | |  | |
| 113 | Where was your residential area? | | 1. 1. Rural 2. 2. Urban | |  | |
| **Part II: Sexual and Reproductive Health-related factors** | | | | | | |
| 114 | | What is your current pregnancy gestational age in weeks? | ------weeks | | |  |
| 115 | | Is it the first pregnancy for you? | 1. 1. Yes 2. 2. No | | | If there is no answer to 116 question |
| 116 | | How often did you become pregnant including the current pregnancy? | ________times including the current pregnancy | | |  |
| 117 | | How often did you give birth/s? | -----births | | |  |
| 118 | | Was there a miscarriage or abortion? | 1. 1. Yes 2. 2. No | | |  |
| 119 | | How much duration of days of your menstruation cycle take? | ------days per cycle | | |  |
| 120 | | How often did you have sexual intercourse per week? | _________ times (days) per week | | |  |
| 121 | | Have you used a contraceptive method? | 1. 1. Yes 2. 2. No | | | If not skip the next. |
| 122 | | What type of contraceptive method did you use? | 1. 1. Condom 2. 2. OC 3. 3. Injection 4. 4. Implant 5. 5. IUD | | |  |
| **Part III: Substance-related personal habit questionnaire** | | | | | | |
| 123 | | Did you drink coffee? | 1. 1. Yes 2. 2. No | If no skip If yes answer the 124 questions. | | |
| 124 | | How many cups of coffee per day? | 1. 1cups/day 2. 2cups/day 3. 3cups/day 4. 4 and above |  | | |
| 125 | | Did your partner drink coffee? | 1. 1. Yes 2. 2. No | If yes answer the 126 questions. | | |
| 126 | | How many cups of coffee per day? | 1. 1. 1cup/day 2. 2. 2cups/day 3. 3. 3cups/day 4. 4. 4 and above cups/day |  | | |
| 127 | | Did you chew khat? | 1. 1. Yes 2. 2. No |  | | |
| 128 | | Did your partner chew khat? | 1. 1. Yes 2. 2. No |  | | |
| 129 | | Did you drink alcohol? | 1. 1. Yes 2. 2. No |  | | |
| 130 | | Did your partner drink alcohol? | 1. 1. Yes 2. 2. No |  | | |
| 131 | | Did you smoke cigarettes? | 1. 1. Yes 2. 2. No |  | | |
| 132 | | If yes how many cigarettes per day? | ____ cigarette /day |  | | |
| 133 | | Did your partner smoke a cigarette? | 1. 1. Yes 2. 2. No |  | | |
| 134 | | If yes how many cigarettes per day? | ____ cigarette /day |  | | |
| **Part IV: Medical-related problem questionnaire** | | | | | | |
| 135 | | Did you know your HIV status before the current pregnancy? | 1. 1. Yes 2. 2. No | If yes answer 136 questions. | | |
| 136 | | What was your HIV status? | 1. 1. Positive 2. 2. Negative 3. 3. Unknown |  | | |
| 137 | | What was your partner's HIV status? | 1. 1. Positive 2. 2. Negative 3. 3. Unknown |  | | |
| 138 | | Have you had any medical problems before the current pregnancy other than HIV? | 1. 1. Yes 2. 2. No 3. 3. Unknown | If not skip to the next | | |
| 139 | | What was it? | 1. 1. Diabetes mellitus 2. 2. Cancer 3. 3. Heart disease 4. 4. Other (specify)__________ |  | | |
| 140 | | Has your partner had a medical problem before the current pregnancy? | 1. 1. Yes 2. 2. No 3. 3. Unknown |  | | |
| 141 | | What was it? | 1. 1. Diabetes mellitus 2. 2. Cancer 3. 3. Heart disease   4. Other (specify)__________ |  | | |
| 142 | | For how long (in months) did you wait to conceive the current pregnancy? | ______ months |  | | |
| **Part V: Lifestyle Factors Questionnaire** | | | | | | |
| 143 | | Have you ever participated in sports/physical activity? | 1. Yes  2. No | If no skip | | |
| 144 | | If yes which type of sport? | 1. Running  2. Jumping rope  3. Riding bicycle  4. Weightlifting  5. Other |  | | |
| 145 | | Have you stressed thinking about some issue? | 1. 1. Yes 2. 2. No | If no skip | | |
| 146 | | If yes, what do you feel stressed/anxious about? | 1. 1. In work 2. 2. In private 3. 3. In love 4. 4. Other |  | | |
| 147 | | Have you (your partner) been exposed to some occupational chemicals and pollutants? | 1. 1. Yes 2. 2. No | If no skip | | |
| 148 | | If yes which of the following? | 1. 1. Radiation 2. 2. Pesticide 3. 3. Welding 4. 4. Other |  | | |

**Thank you for participating in this study!!!**
